# Dr. Charles Albert Young

**Published:** 1911-08-26

**Authors:** 


Dr. Charles Albert Young, late of Hertford, has died at Atherstone, Warwickshire. He was a B.A. of Cambridge University and a student at St. Bartholomew's Hospital, becoming qualified in London in 1893 with the conjoint diploma. He held for some time the appointment of resident medical officer at the Hertford County Hospital.

